# Using a Multisectoral Approach to Advance Health Equity in Rural Arizona: Community-Engaged Survey Development and Implementation Study

**DOI:** 10.2196/25577

**Published:** 2021-05-12

**Authors:** Mark Remiker, Samantha Sabo, Dulce Jiménez, Alexandra Samarron Longorio, Carmenlita Chief, Heather Williamson, Nicolette Teufel-Shone

**Affiliations:** 1 Center for Health Equity Research Northern Arizona University Flagstaff, AZ United States

**Keywords:** health equity, community-engaged, multisector, survey development

## Abstract

**Background:**

Over the past decade, public health research and practice sectors have shifted their focus away from identifying health disparities and toward addressing the social, environmental, and economic determinants of health equity. Given the complex and interrelated nature of these determinants, developing policies that will advance health equity requires collaboration across sectors outside of health. However, engaging various stakeholder groups, tapping into their unique knowledge systems, and identifying common objectives across sectors is difficult and time consuming and can impede collaborative efforts.

**Objective:**

The Southwest Health Equity Research Collaborative at Northern Arizona University, in partnership with an 11-member community advisory council, is addressing this need with a joint community-campus effort to develop and implement a Regional Health Equity Survey (RHES) designed to generate an interdisciplinary body of knowledge, which will be used to guide future multisectoral action for improving community health and well-being.

**Methods:**

Researchers and community partners used facilitated discussions and free listing techniques to generate survey items. The community partners pilot tested the survey instrument to evaluate its feasibility and duration before survey administration. Respondent-driven sampling was used to ensure that participants included leadership from across all sectors and regions of northern Arizona.

**Results:**

Over the course of 6 months, 206 participants representing 13 sectors across the 5 counties of northern Arizona were recruited to participate in an RHES. Survey response rates, completion percentage, and sector representation were used to assess the effectiveness and feasibility of using a community-engaged apporach for survey development and participant recruitment. The findings describe the current capacity to impact health equity by using a multisectoral approach in northern Arizona.

**Conclusions:**

The Southwest Health Equity Research Collaborative effectively engaged community members to assist with the development and implementation of an RHES aimed at understanding and promoting multisectoral action on the root causes of health inequity. The results will help to build research and evaluation capacity to address the social, economic, and environmental conditions of health inequity in the region.

## Introduction

### Background

Over recent decades, eliminating health disparities has been a major focus of public health efforts in the United States [1,2]. The social determinants of health (SDOH) framework is often used to guide health disparities research by defining the conditions in which people are born, grow, live, work, and age and demonstrating how these factors differentially shape health outcomes within and between populations. Although health disparities and SDOH approaches have offered valuable insights into the conditions and contexts that contribute to sickness and wellness among specific populations, these concepts are limited because they do not expose the important pathways by which social identity (eg, race and gender), the distribution of power and resources, and institutional policies shape opportunities for health. More recently, to address the underlying social inequalities that lead to differential health outcomes across population groups, public health research has shifted its focus toward a health equity framework [3-5].

In 2013, the Robert Wood Johnson Foundation launched a nationwide health equity effort called the *Culture of Health Initiative*, which aimed at making health a shared value; fostering cross-sector collaboration to achieve well-being; and creating healthier, more equitable communities [6]. Health equity initiatives have also been incorporated at the federal level in the United States through the creation of Offices of Minority Health and the goals of Healthy People 2020, which focus on achieving health equity by eliminating disparities and improving the health of all groups [7]. Despite these worthwhile efforts, health disparities and health inequities still loom large in the United States, particularly for people of color and rural communities [8,9].

The barriers to effective action on health equity may be due, in part, to a lack of intersectoral collaboration and consensus on how to identify and overcome the root causes of health inequity, which is defined as the underlying social, economic, and environmental inequalities that create different living conditions among and between populations. A multisectoral approach (MSA) to addressing health equity refers to “deliberate collaboration among various stakeholder groups and sectors (eg, public health, transportation, education, criminal justice) to jointly achieve a policy outcome” [10]. Using an MSA to improve health equity can have multiple benefits, including pooling resources, leveraging unique knowledge bases, expanding reach, and avoiding the duplication of work. This approach is highlighted in the *Health in All Policies* framework, which engages cross-sectoral partners in the promotion of health equity while simultaneously advancing other goals, such as promoting job creation and economic stability [11].

### Objective

A major contributor to the lack of successful cross-sectoral collaboration is the problematic perception that addressing issues related to health equity is the sole responsibility of those working in health-related fields [12]. However, given that the root causes of health inequity are diverse, complex, evolving, and interdependent in nature [13], making progress toward health equity will require collaboration across sectors [3,14].

To address this fundamental issue, we describe the community-engaged development and implementation of the Northern Arizona University (NAU), Southwest Health Equity Research Collaborative’s (SHERC) Regional Health Equity Survey (RHES) [15]. The RHES is designed to understand and strengthen research, practice, policy, and organizational capacity to address locally identified health equity issues using an MSA.

The SHERC is a National Institute of Health–funded Research Centers in Minority Institution (RCMI) initiative of the Center for Health Equity Research (CHER) at NAU. The overall goal of the SHERC is to increase basic biomedical, clinical, and behavioral research at NAU to address health disparities among diverse populations of the southwestern United States. The SHERC consists of 5 cores that interact synergistically: administration, investigator development, recruitment, research infrastructure, and community engagement. This paper focuses on the community engagement core’s (CEC) efforts to address collaborative engagement in health equity in northern Arizona.

The CEC endeavors to cultivate and sustain productive collaborations and partnerships with community-based organizations and leaders in meaningful ways that foster awareness and participation in health equity research. Broadly, the CEC is guided by the *Communities in Action–Pathways to Health Equity Model* [3] grounded in the Robert Wood Johnson *Culture of Health Action Framework* and the Prevention Institute’s *Framework of Emerging Systems to Achieve an Equitable Culture of Health* [6]. These asset-based frameworks recognize that health is impacted by multiple social determinants and that health inequity is produced by multilevel systems such as poverty, structural racism, and discrimination. Therefore, community-based solutions are necessary, but not sufficient, to achieve health equity.

The main objective of the CEC is to engage community-based organizations, community leaders, policy experts, and researchers from various sectors, including childhood development, education, criminal justice, public health, and policy, to (1) identify commonalities in health trends and social, structural, and environmental factors that contribute to health inequity and (2) understand and strengthen organizational capacity to address locally identified health equity issues using an MSA. A primary step in defining public health priorities and understanding the community’s current capacity to impact health inequities is through the systematic collection of information [16].

As a newly established research center, CHER took the first step to better understand public health priorities in 2017 through a collaboration with regional partners, which produced the groundbreaking Regional Health Equity Assessment (RHEA) [17]. Unique in its breadth and scope, the RHEA engaged more than 100 regional thought leaders in interviews, forums, and conferences; synthesized 57 community needs assessments; and produced a local analysis of public health data sets to prioritize health equity goals for northern Arizona. Building on the highly participatory work of the RHEA, and through new funding, the SHERC CEC launched the 2020 RHES designed to take the next step of understanding and strengthening organizational capacity to address locally identified health equity issues in northern Arizona using an MSA.

## Methods

### Regional Overview

**Figure 1:**
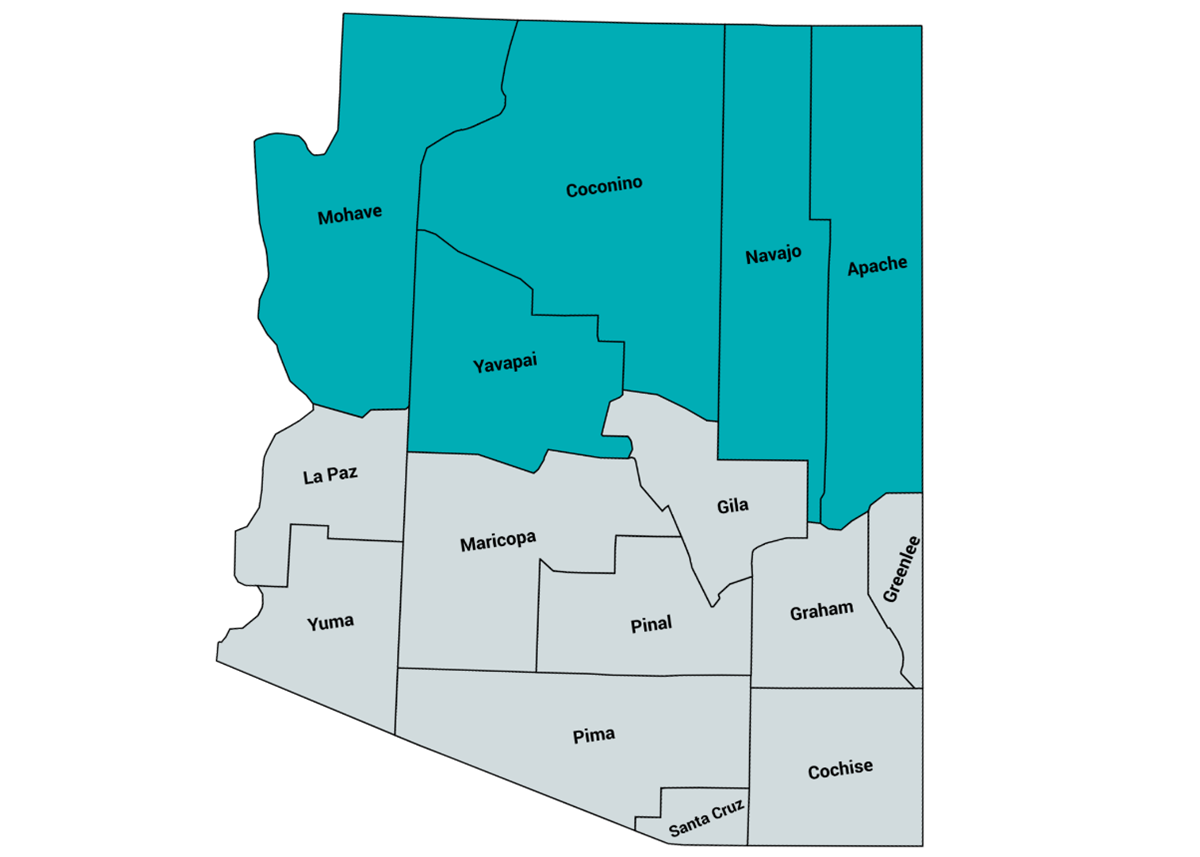
Map of northern Arizona counties.

Geographically positioned atop the Colorado Plateau, the northern Arizona region covers more than 6000 square miles of land; is home to 12 federally recognized American Indian tribes; and comprises the following 5 counties: Apache, Coconino, Mohave, Navajo, and Yavapai (Figure 1). This region is also characterized by great cultural and ethnic diversity. As of 2017, 62.5% of the people in northern Arizona identified as White, 22.5% identified as American Indian, and 11% identified as Hispanic [17]. With 37% of the residents living in areas with a population of less than 2500 people, the northern Arizona region is largely rural [17]. In 2018, over 20% of the population was living in poverty, and the per capita annual income was US $22,636—more than US $30,000 less than the median household income of the entire state of Arizona [18]. The counties in northern Arizona vary greatly in demographics, such as ethnicity and age, and in degrees of access to all types of services, opportunities, and utilities necessary for healthy people and communities. This diversity makes it important to consider health issues as well as community assets and challenges in a locally specific context and makes this region a scientifically significant venue for the protocol identified in this paper.

### Community Advisory Council

Given the diversity of the northern Arizona region, it is crucial that any initiatives addressing health inequity be community driven. Community advisory councils (CACs) can benefit research institutions by ensuring that the research agenda aligns with priorities that are salient within the community. In addition to providing their unique perspectives and expertise to guide the development of research projects, CAC members can help to bridge gaps and build trust between the community and the research institution [19]. Early on, the CEC established an 11-member CAC composed of leaders from distinct sectors important to achieving health equity across northern Arizona, including early childhood development, education, criminal justice, public health, and policy. Researchers and CAC members met face-to-face and remotely throughout the survey development and implementation process.

### Survey Development and Implementation

**Figure 2:**
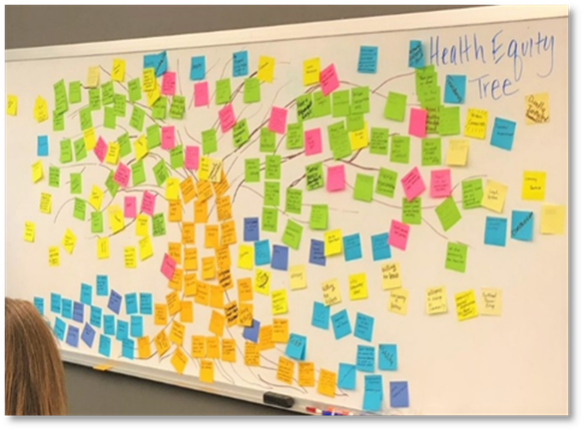
Community advisory council free-listing activity.

The initial stage of the survey development occurred in April 2018 with a half-day, in-person survey workshop between personally, professionally, and geographically diverse members of the CAC and the CEC. The RHES workshop was guided by meaningful learning theory and popular education techniques, which acknowledge that adult learners integrate new knowledge into what is already known and create a cognitive structure to make sense of their surroundings and situations [20,21]. After an introduction to the overarching goals of the RHES, participants in the CAC and CEC engaged in a facilitated discussion to collectively acknowledge operating systems of power and privilege and define the *root causes* of health inequity in our region. Working from this common understanding of perceived and experienced challenges to health equity in our region, members of the CAC and CEC participated in a free-listing activity aimed at generating specific items for the RHES. Free listing is a technique used to gather data about a specific domain or topic by asking people to list all the items they can think of related to the topic. By using sticky notes in the corresponding color and shape of components of a robust and healthy tree, we identified the specifics of the 5 primary RHES constructs important to achieve, maintain, and scale health equity in our region: outcomes (leaves), innovations (flowers and fruits), measurement (tree bark), sustainability (rain and sunshine), and partnerships (bees and other cross-pollinators). Figure 2 shows an image of the tree activity. The CEC research staff then transcribed the responses and sorted them into categories within the 5 major RHES constructs. The constructs identified by the CAC were combined with other existing health equity assessments (eg, the Bay Area Regional Health Inequity Initiative Organizational Self-Assessment) [22] to generate the initial set of RHES constructs and questions.

The survey questions underwent 2 rounds of edits by CAC members, SHERC project staff, and SHERC research leadership. The final RHES is composed of 48 open- and closed-ended questions covering topics including, but not limited to, the extent and focus of the current cross-sectoral partnerships, priority areas for future research, and the use of data in decision making. To aid participants in completing the survey, the CEC added a glossary of definitions and examples of major public health concepts such as *health disparity*, *SDOH*, and *root causes of health inequity*. The final RHES was generated using Qualtrics [23], a web-based survey platform, and pilot tested by the CEC staff and members of the CAC.

The population of interest for the RHES included community, organizational, and grassroots leaders from the 5 aforementioned northern Arizona counties. In line with the Vitalyst Health Foundation’s elements of a healthy community [24], our sectors of interest included (1) community health and economic development; (2) health and human services; (3) law, justice, and public safety; (4) parks and recreation; (5) policy; (6) early childhood development; (7) transportation; (8) food systems; (9) housing; (10) education; (11) arts, music, and culture; (12) planning and zoning; and (13) cultural resources management.

A 3-pronged approach was used to identify potential participants for the RHES. First, extensive internet searches were conducted to identify individuals in positions of leadership across sectors and counties of northern Arizona. Second, the CAC members nominated leaders from their regions and sectors. Finally, the CEC staff presented the RHES and circulated RHES sign-up sheets at county-level leadership meetings attended by the target population. Attendees were encouraged to add the names of sector leaders who were not present at the meeting. All potential participants’ names were compiled, duplicate names were removed, and county-level participant lists were generated for each sector. Before administering the RHES, at least two county champions (eg, assistant county manager and local public health director) vetted each county’s list, removing names of individuals who were no longer in their positions and filing in gaps in sectors where there was no representation.

**Figure 3:**
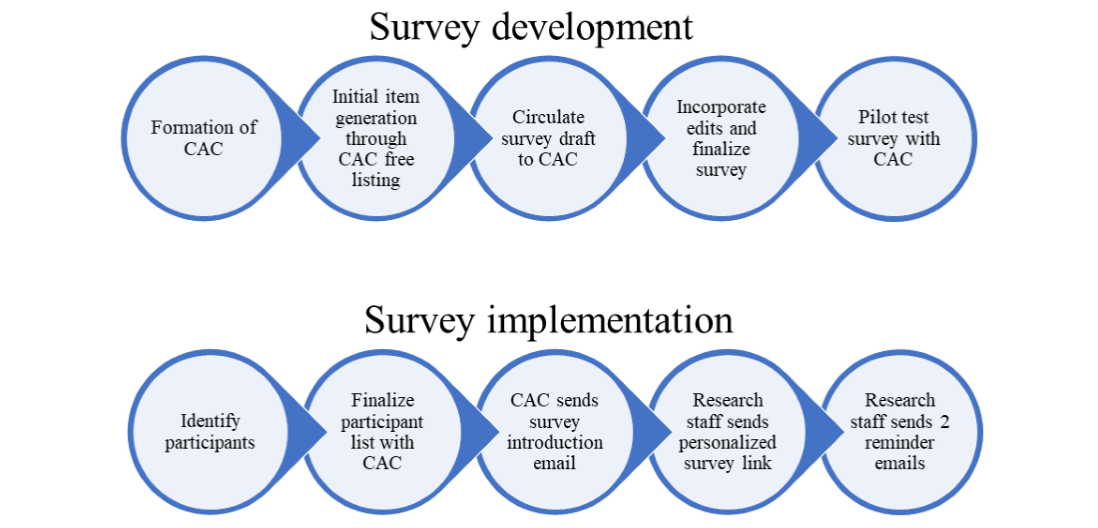
Regional Health Equity Survey development and implementation flow chart. CAC: community advisory council.

Once participant lists were finalized, introductory emails were sent by the county champions, alerting all potential participants of the RHES, followed by a personalized email with links to the survey 1 day after the introductory emails were sent. Participants received 2 reminder emails 2 and 4 weeks after the initial invitation. All respondents were offered a US $25 gift card as compensation for their participation. Figure 3 illustrates the survey development and implementation process.

### Data Analysis

All descriptive statistics were analyzed using IBM SPSS (version 26). Depending on the responses, qualitative data from open-ended questions were analyzed using either a priori coding or emergent coding and a thematic analysis approach (ATLAS.ti 8, Scientific Software Development GmbH). The Vitalyst Health Foundation’s elements of a healthy community [24] were applied to questions where the data were suited for a priori coding. Data were coded by one researcher, and consensus on codes and themes was achieved through intensive discussion with a second researcher throughout the analysis process.

### Human Participant Compliance

The development and implementation of the RHES were reviewed and deemed nonhuman subjects research by the NAU’s institutional review board (project number: 1198096-1).

## Results

### Participant Demographics

A total of 206 of the 560 invited multisectoral representatives (response rate 37%) from northern Arizona participated in the RHES. Of those who participated, 64.1% (132/206) completed the entire survey, whereas 27.6% (57/206) answered 30% or less of the survey questions. Table 1 provides a summary of the survey respondents’ demographics.

Although there was a relatively equal distribution across genders with all counties combined (female: 69/129, 53.4%; male 56/129, 43.4%), there was very little ethno-racial diversity, as most of the respondents identified as White (108/129, 83.7%). Survey results indicated that respondents were well established in their sectors (Table 1). On average, participants reported working in their respective sector for over 199.4 (SD 133.3) months, and had been at their current position for an average of 63.7 (SD 71.7) months.

Half of the participants (102/204, 50%) held government positions at the federal, state, county, and municipality levels, and approximately one-third of respondents (45/135, 33%) said that they had an active role or were the primary decision maker within their organization. The reported leadership positions of participants included, but were not limited to, county managers and department directors, chief of police, superintendents, presidents, chief executive officers, and executive directors. Most participants reported either working directly with community members (154/192, 80%) or supervising staff who worked directly with community members (140/192, 73%).

**Table 1:** Participant demographics by county.

Characteristics		County					
		Apache (n=8)	Coconino (n=94)	Mohave (n=34)	Navajo (n=28)	Yavapai (n=42)	Total (N=206)
**Gender, n (%)**							
	Female	1 (13)	20 (21)	16 (47)	8 (29)	24 (57)	69 (53)
	Male	4 (50)	26 (28)	7 (21)	11 (39)	8 (19)	56 (43)
	Other	0 (0)	0 (0)	0 (0)	0 (0)	1 (2)	1 (1)
	No answer	0 (0)	1 (1)	0 (0)	2 (7)	0 (0)	3 (2)
**Race and ethnicity, n (%)**							
	American Indian or Alaskan Native	0 (0)	2 (2)	0 (0)	1 (4)	0 (0)	3 (2)
	Asian or Pacific Islander	0 (0)	0 (0)	1 (3)	0 (0)	0 (0)	1 (1)
	Black or African American	0 (0)	3 (3)	0 (0)	0 (0)	0 (0)	3 (2)
	Hispanic or Latino	0 (0)	2 (2)	0 (0)	0 (0)	4 (10)	6 (5)
	White	5 (62)	40 (43)	19 (56)	17 (61)	27 (64)	108 (84)
	Other	0 (0)	0 (0)	1 (3)	0 (0)	2 (5)	3 (2)
	No answer	0 (0)	0 (0)	2 (6)	3 (11)	0 (0)	5 (4)
Age (years), mean (SD)		52.6 (5.9)	45.8 (10.1)	52.7 (11.1)	50.9 (9.7)	49.4 (14.4)	49 (11.6)
Position time (months), mean (SD)		21.1 (15.6)	58.1 (71.9)	79.8 (78.5)	69.9 (59.2)	65.4 (77.2)	63.7 (71.7)
Sector time (months), mean (SD)		91.1 (87)	192.4 (116.2)	233.6 (147.3)	480 (199.6)	204.2 (150.8)	199.4 (133.3)

In some of the less populated counties, individuals may be responsible for leading multiple departments; thus, participants could identify with more than one sector. Of the respondents, approximately 71.8% (148/206) identified with only 1 sector. Although there was representation from all 13 sectors, most participants identified in part with either *health and human services* (95/206, 46.1%); *education* (48/206, 23.3%); *community and economic development* (34/206, 16.5%); or *law, justice, and public safety* (34/206, 16.5%). A majority of sectors had representation from each county; however, there were 6 instances where 1 county had no sector representation. For instance, no leadership from Yavapai county identified with the *arts, music, and culture* sector. Table 2 shows the distribution of sector by county representation among survey respondents.

**Table 2:** Distribution of participants by county and self-identified sector.

Participant self-identified sector	County					
	Apache (n=8), n (%)	Coconino (n=94), n (%)	Mohave (n=34), n (%)	Navajo (n=28), n (%)	Yavapai (n=42), n (%)	Total (N=206), n (%)
Health and human services	2 (25)	42 (45)	14 (41)	11 (4)	26 (62)	95 (46)
Education	1 (13)	18 (19)	11 (32)	7 (25)	11 (26)	48 (23)
Community and economic development	1 (13)	13 (14)	7 (21)	6 (21)	7 (17)	34 (17)
Law, justice, and public safety	2 (25)	21 (22)	0 (0)	5 (18)	6 (14)	34 (17)
Policy	1 (13)	13 (14)	3 (9)	1 (4)	7 (17)	25 (12)
Housing	0 (0)	11 (12)	1 (3)	2 (7)	4 (10)	18 (9)
Transportation	1 (13)	6 (6)	3 (9)	3 (11)	4 (10)	17 (8)
Food systems	1 (13)	1 (1)	6 (18)	1 (4)	4 (10)	13 (6)
Early childhood development	0 (0)	4 (4)	4 (12)	1 (4)	3 (7)	12 (6)
Parks and recreation	1 (13)	4 (4)	2 (6)	3 (11)	1 (2)	11 (5)
Planning and zoning	1 (13)	6 (6)	0 (0)	1 (4)	1 (2)	9 (4)
Arts, music, and culture	1 (13)	3 (3)	1 (3)	1 (4)	0 (0)	6 (3)
Cultural resource management	1 (13)	1 (1)	0 (0)	2 (7)	1 (2)	5 (2)

### Cross-Sectoral Partnerships

**Figure 4:**
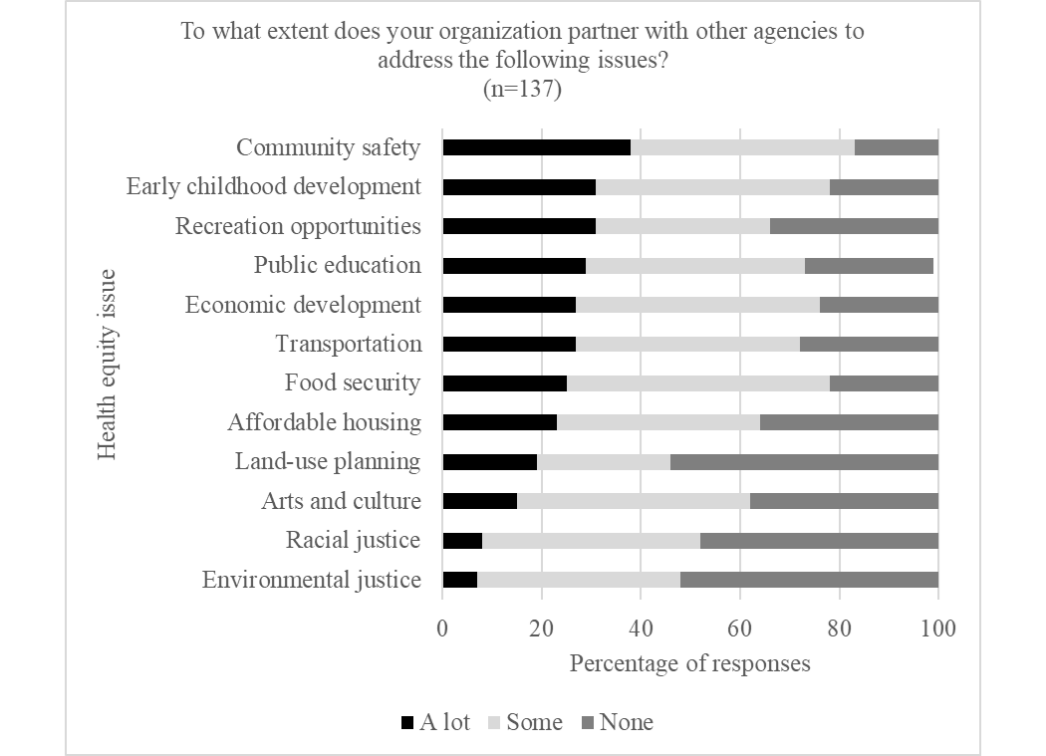
Extent of cross-sectoral collaborations on health equity issues.

The most frequently cited characteristics for developing a successful multisectoral partnership were communication, shared vision, and trust*.* Approximately 74.7% (130/174) of participants reported that they had previously partnered with a different sector to address environmental, social, and economic conditions that impact health, and 94.6% (123/130) of those individuals indicated a past partnership with more than 1 sector (average number of partnerships 6.7, SD 3.5). As displayed in Figure 4, *community safety*, *early childhood development*, and *recreation opportunities* were the primary issues on which organizations most often collaborated with other sectors, whereas *land-use planning*, *racial justice*, and *environmental justice* were the issues least likely to garner cross-sectoral attention.

### Role of Research

To examine how research can effectively influence health equity in northern Arizona, participants were asked, “What role do you think research has in addressing the environmental, social, and economic conditions that impact health in the community you serve?” Most leaders asserted that research plays a significant role in addressing the root causes of health inequity, whereas very few participants felt the role of research was *little* or *none*. Often, participants described the limitations of research, expressing that although research plays an important role in identifying, understanding, and addressing needs or problems in their communities, the right conditions must be met, including conducting research responsibly and ethically, using scientifically sound methods, and yielding actionable results to directly and positively impact the community. Specifically, leaders believed that research affords an opportunity to illuminate and understand the gaps and problems experienced by the community and serves to validate the community’s knowledge and lived experiences of their own needs; leaders further indicated that this information can be used for action. Textbox 1 outlines the areas of research that were identified by community leaders as a priority for future studies on health equity.

**Community-identified priority areas for health equity research.**

**Area of research and specific research topics**

- Economic opportunities: Poverty, disparities in income, job opportunity and lack of higher wage jobs, workforce development, economic development, and economic indicators
- Health care: Access, affordability, and quality of health services and health plan coverage; long distances people must travel to seek care; and understaffing and difficulty attracting and retaining health care professionals, especially in rural areas
- Behavioral health: Access to mental health and substance use services, including drug addiction, rehabilitation; addressing stigma related to mental health and substance use
- Education: Educational opportunities from kindergarten through high school through higher education, affordability, and funding
- Transportation: Access, affordability, and adequacy
- Housing: Access, affordability, and homelessness
- Food: Access, food security, and quality or healthy foods
- Early childhood: Early childhood education and youth development
- Social context: Social context around health inequities, understanding issues around culture, stigma related to health conditions, and social activities
- Social justice: Effects of incarceration, historical trauma, and social justice in relation to other social determinants of health
- Environment: Climate change
- Tribal communities: Funding, focus and effectiveness of Indian Health Services, health care options on the reservation, and impact of native American culture on health maintenance
- Rural communities: Access to services based on unique challenges experienced by rural communities
- Aging and older people: Access to services

### Use of Data in Decision Making

**Figure 5:**
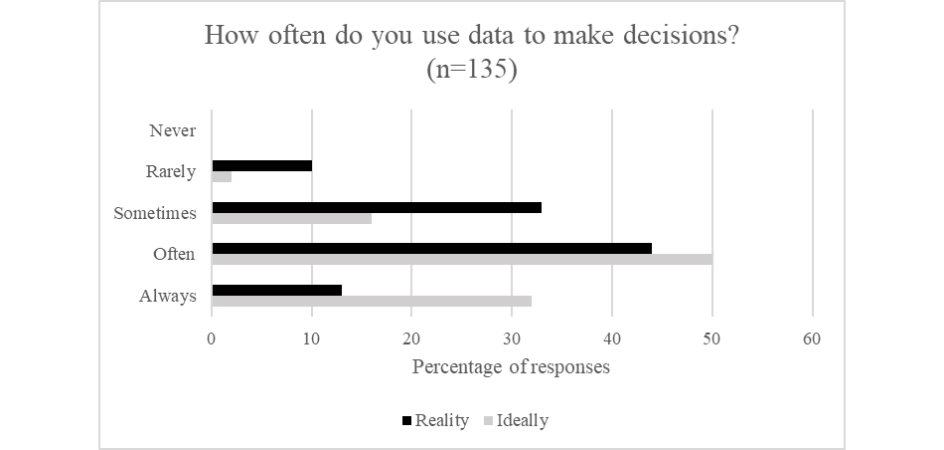
Use of data for decision making.

Data-informed health promotion is an emerging and ever-changing theme in public health research and practice. To examine the current data use across sectors, leaders were asked how often they used data to make decisions and what barriers they faced in the process. Across all leaders, 93.3% (126/135) reported having used data to make decisions; however, there exists a gap between how often data are currently used and how often leaders would ideally use data to guide their decision making (Figure 5). Although 81.4% (110/135) of sector leaders would prefer to *always* or *often* use data to make decisions, only 56.2% (76/135) currently use data to make decisions. This pattern emerged for participants who identified with either a single sector or more than 1 sector. Furthermore, over 60.7% (82/135) of the respondents said that they did encounter barriers to using data to make decisions. When asked to identify the biggest barriers to using data, leaders most often cited a lack of useful available data, followed by an absence of the expertise needed to analyze the data.

## Discussion

### Principal Findings

In this paper, we describe how the SHERC at NAU effectively engaged community members to assist in the development and implementation of the RHES, with the goal of understanding and promoting multisectoral action on the root causes of health inequity in northern Arizona. Furthermore, we demonstrate how over 200 county-level leaders from various sectors, beyond public health and health care, were recruited to share their knowledge, attitudes, and actions to address the social, environmental, and economic conditions that impact health and well-being in the region.

Overall, our work revealed that using a community-engaged approach to survey local leadership can be an effective first step toward identifying and prioritizing areas of action on the root causes of health inequity. Specifically, using a community-engaged approach to develop and implement the RHES ensured that (1) survey questions resonated with community priorities and (2) respondents were recognized as representatives of their community. With participation across all sectors and counties of interest (Table 2) and an average reported time of 16.6 years working in their sector, we are confident that the outcomes of the RHES capture the perspectives of multisectoral leadership in northern Arizona and reveal the range of factors that contribute to health inequity in the region.

This study also exposes the benefits and challenges of developing and implementing cross-sectoral partnerships to address health inequities. Although collaboration with governmental and nongovernmental sectors outside of health is required to develop policies and programs to advance health equity, establishing and maintaining effective cross-sectoral partnerships is not an easy task [25]. For example, engaging relevant stakeholders, tapping into their unique knowledge systems, and identifying common objectives across sectors requires time and resources that are not often afforded to governmental and nonprofit agencies. As the local *anchor institution* and RCMI in northern Arizona, NAU is in a unique position to leverage their human resources and expertise to collect and disseminate cross-sectoral perspectives on regional issues of health inequity [3]. The approaches and methods used in this study serve as a model for other universities and RCMIs, in particular, to advance community-driven health equity agendas.

### Implications for Research and Practice

Findings from this study suggest that county-level leaders in northern Arizona are currently working across sectors to address the root causes of health inequity; however, the extent to which they partner is limited, and the issues being addressed are bounded and unbalanced (Figure 3). These results indicate that there is capacity to impact health inequities using an MSA, but this work could benefit from more deliberate coordination across sectors. Recent research by Narain et al [26] shows that framing issues of health equity in ways that resonate with sectors outside of public health was valuable for promoting cross-sectoral work to improve health equity. Focusing on the cross-sectoral research priorities that were identified in the RHES (Textbox 1) can, therefore, help to determine promising areas for collaborative action in northern Arizona.

Results from the RHES further indicated that data-driven decision making is highly valued among participating leaders, but most found data to be outdated or unavailable or they worked in an environment in which expertise to analyze data was lacking. Lack of access to health-related data is particularly salient in rural areas, such as northern Arizona [27]. Consequently, differential access to data has the potential to perpetuate and exacerbate health inequities in rural areas. As a local academic institution with expertise in methods of data collection and analysis, NAU is poised to collaborate with community partners to improve their ability to access and use high-quality data to inform decisions regarding health equity. The NAU SHERC has already taken steps toward this goal by inviting community partners to participate in monthly research methods workshops.

Finally, this study illustrated the potential utility of using a baseline assessment of organizational leaders to start a productive dialog on the various and unique ways in which each sector (eg, housing, transportation, justice, economic development, education, arts, and culture) can strengthen the health and well-being of their community. Following the Bay Area Regional Health Inequities Initiative framework [22], the RHES results will be used to guide research priority areas and practice and policy efforts of the SHERC, CHER, and NAU as a whole. Our next steps include (1) engaging our scientific and community advisory boards in the further interpretation of results and recommendations for research, practice, and policy; (2) hosting a series of regional community events to share survey results and dialog on the strategic next steps toward developing a regional health equity initiative that meaningfully engages stakeholders across sectors; and (3) promoting further community-campus collaboration through the development of community engagement studios [28].

### Limitations

Although a community-engaged approach to survey development for health disparities research has clear benefits [16], it is important to recognize that it also comes with unique challenges. For example, it is difficult to develop a comprehensive yet concise survey on health equity that includes the diverse voices of the community and limits the burden on the participant. In this study, 27.6% (57/206) of leaders who started our survey answered only 30% or less of the survey questions, indicating that survey length may have been a deterrent for some individuals. Furthermore, respondent-driven sampling methods were used to recruit participants for the RHES, whereby county leaders were asked to nominate other colleagues to complete the survey. Although this approach helped us to compile a comprehensive list of local leaders, the findings are specific to the region of northern Arizona and should not be generalized to other areas of the United States. However, we believe that our survey development and implementation procedures can be used as a model for other institutions to conduct similar efforts in their communities. Importantly, we also acknowledge the lack of racial and ethnic diversity in our respondents; however, we are uncertain if this is a limitation of our recruitment strategy or a true reflection of the lack of diversity in leadership in northern Arizona. Future studies can examine the health priorities among the diverse community members of northern Arizona to ascertain if their health priorities align with the priorities of those in leadership roles.

### Conclusions

Without a clear consensus on the root causes of health equity and greater cross-sectoral collaboration, the development of effective policy and practice objectives aimed at reducing health disparities and improving health equity will be limited [29]. As anchor institutions, local universities are well positioned to help lead multisector work aimed at eliminating health disparities and making advancements in the promotion of health equity [3]. In this study, we outlined the steps for engaging multisectoral leaders in survey development and distribution as a promising first step toward developing meaningful multisectoral collaborations across a diverse region of the southwestern United States.

## Abbreviations

CAC: community advisory council
CEC: community engagement core
CHER: Center for Health Equity Research
MSA: multisectoral approach
NAU: Northern Arizona University
RCMI: Research Centers in Minority Institution
RHEA: Regional Health Equity Assessment
RHES: Regional Health Equity Survey
SDOH: social determinants of health
SHERC: Southwest Health Equity Research Collaborative
